# Development of Two-Layer Hybrid Scaffolds Based on Oxidized Polyvinyl Alcohol and Bioactivated Chitosan Sponges for Tissue Engineering Purposes

**DOI:** 10.3390/ijms232012059

**Published:** 2022-10-11

**Authors:** Elena Stocco, Silvia Barbon, Elena Zeni, Leonardo Cassari, Annj Zamuner, Antonio Gloria, Teresa Russo, Rafael Boscolo-Berto, Maria Martina Sfriso, Veronica Macchi, Raffaele De Caro, Monica Dettin, Andrea Porzionato

**Affiliations:** 1 Deparment of Neuroscience, Section of Human Anatomy, University of Padova, 35121 Padova, Italy; 2 L.i.f.e.L.a.b. Program, Consorzio per la Ricerca Sanitaria (CORIS), Veneto Region, 35128 Padova, Italy; 3 Department of Industrial Engineering, University of Padova, 35122 Padova, Italy; 4 Institute of Polymers, Composites and Biomaterials, National Research Council of Italy, 80125 Naples, Italy; 5 Department of Industrial Engineering, University of Naples Federico II, 80125 Naples, Italy

**Keywords:** oxidized polyvinyl alcohol, chitosan sponges, mechanical analysis, hybrid scaffolds, self-assembling peptides, peripheral nerve injury, nerve regeneration, nerve conduits

## Abstract

Oxidized polyvinyl alcohol (OxPVA) is a new polymer for the fabrication of nerve conduits (NCs). Looking for OxPVA device optimization and coupling it with a natural sheath may boost bioactivity. Thus, OxPVA/chitosan sponges (ChS) as hybrid scaffolds were investigated to predict in the vivo behaviour of two-layered NCs. To encourage interaction with cells, ChS were functionalized with the self-assembling-peptide (SAP) EAK, without/with the laminin-derived sequences -IKVAV/-YIGSR. Thus, ChS and the hybrid scaffolds were characterized for mechanical properties, ultrastructure (Scanning Electron Microscopy, SEM), bioactivity, and biocompatibility. Regarding mechanical analysis, the peptide-free ChS showed the highest values of compressive modulus and maximum stress. However, among +EAK groups, ChS+EAK showed a significantly higher maximum stress than that found for ChS+EAK-IKVAV and ChS+EAK-YIGSR. Considering ultrastructure, microporous interconnections were tighter in both the OxPVA/ChS and +EAK groups than in the others; all the scaffolds induced SH-SY5Y cells’ adhesion/proliferation, with significant differences from day 7 and a higher total cell number for OxPVA/ChS+EAK scaffolds, in accordance with SEM. The scaffolds elicited only a slight inflammation after 14 days of subcutaneous implantation in Balb/c mice, proving biocompatibility. ChS porosity, EAK 3D features and neuro-friendly attitude (shared with IKVAV/YIGSR motifs) may confer to OxPVA certain bioactivity, laying the basis for future appealing NCs.

## 1. Introduction

Neurons’ limited ability in self-regeneration is a significant issue in case of severe peripheral nerve injuries (PNIs). To date, despite advances in microsurgical techniques, peripheral nerve recovery remains a challenge and complete sensory/motor function restoration has never been achieved [1]. Hence, to support and guide a successful axonal nerve growth without resorting to autologous grafts (limited availability, comorbidities) or allografts/xenografts (possible immunological reactions), “on-the-bench” nerve conduits (NCs) have long been investigated [2]. Currently, the Food and Drug Administration (FDA) has approved 11 natural (collagen type I)/synthetic (polyglycolic acid, poly(D,L-lactide-co-ε-caprolactone), polyvinyl alcohol) polymer-based conduits for PNIs repair but in vivo outcomes are still not fully satisfactory, thus encouraging further research and development in this field [3].

Vanguard NCs are not merely pipes connecting the opposing stumps of a severed nerve. They are thought to be highly bioactive mimicking the natural extracellular matrix (ECM) environment, boosting axons’ number and regeneration speed and length, while discouraging fibroconnective tissue formation and infiltration. Additionally, also a proper biodegradation rate is pursued to avoid a second surgery after implantation [3,4]. To meet these clinical needs, Tissue Engineering (TE), including focused in-materials research, represents significantly valuable resources, allowing the build-up of functional and customizable devices displaying a proper balance among biological, mechanical, and physical features [5,6].

Within the broad panorama of materials adopted in TE, the fundamental requirements they are expected to show include biocompatibility and biodegradability, appropriate mechanical integrity and porosity, and processability and manipulability. Considering the fabrication of NCs, conductivity and transparency may be also looked for to specifically meet the environment and the surgery specificities [7]. Typically, synthetic materials are appealing due to high reproducibility, easier large-scale production, and tuneable mechanical properties. However, they may suffer from low biological activity and eventual cytotoxicity associated with biodegradation molecules release. In parallel, natural materials are highly bioactive and biocompatible; they are easily degraded and metabolized by host tissues without eliciting adverse reactions. Unfortunately, specific limitations include the need for extensive purification and batch-to-batch variability affecting mechanical/biological properties and degradation rate [5]. To overcome the peculiar restrictions associated with both synthetic and natural polymers, the fabrication of composite/hybrid devices, including NCs, may serve as a smart alternative, counterbalancing and controlling the specific drawbacks and leading to a customized and promising device [8,9,10].

Focusing on synthetic materials, a new and versatile biomaterial for scaffold preparation, oxidized polyvinyl alcohol (OxPVA), was recently synthetized and described [11,12,13]. Compared to its native counterpart, OxPVA is endowed with attractive specificities, including a tuneable biodegradation rate, highly potential bioactivity, and adjustable physical characteristics. Preclinical studies in animal models of PNI-confirmed OxPVA wraps/NCs tempting features [14,15]; however, further in-depth studies are still required to gain awareness and full control over OxPVA potentialities. For instance, intense efforts are devoted towards the identification of smart bioactivation strategies promoting the cell–polymer interactions [16,17]. When looking for the optimization of OxPVA NCs’ microenvironment, coupling it with a natural sheath may be appealing. To date, decellularized ECM-OxPVA combinations have been promising revelations for different end-use destinations, including cartilage [9] and short bowels [10]; thus, its matching with natural polymers may be also useful.

Chitosan is the deacetylated form of chitin, the constituent of the crustaceans’ exoskeleton. It is a linear polysaccharide formed by glucosamine and N-acetyl glucosamine units, linked by β (1–4) glycosidic bonds. Chitosan is soluble in slightly acidic medium, which, together with its cationic nature, makes it particularly attractive in TE, also due to its ability to form sponges [2]. Referring to PNI recovery, it can support axon regrowth while reducing fibroconnective tissue formation; additionally, chitosan biodegradation products (chito-oligosaccharides) can promote nerve regeneration [18,19].

In consideration of these appealing characteristics, here we aimed to test the in vitro bioactivity and in vivo biocompatibility of hybrid scaffolds combining OxPVA and chitosan sponges (ChS) to predict the therapeutic behaviour of complex two-layered scaffolds prepared as tubular NCs for peripheral nerve regeneration. Additionally, to further encourage the scaffolds bioactivity, ChS bioactivation with the self-assembling-peptides (SAP) occurred. SAPs are hydrogels generated by simple molecules organized in nano- or micro-metric structures supporting the exchange of bioactive factors, oxygen, nutrients, and waste products among cells and the environment [20]. In this work, the SAP EAK, eventually bound to the IKVAV (EAK-IKVAV) or YIGSR (EAK-YIGSR) laminin-derived adhesive sequences, were considered. ChS porosity (ultrastructure), together with EAK 3D intrinsic features and neuro-friendly attitude also shared with the IKVAV and YIGSR motifs, may confer to OxPVA a certain bioactivity, thus laying the basis for future appealing NCs to fully respond and satisfy both surgeons’ and patients’ expectations. Furthermore, heterotopic implant-based evidence also confirmed in vitro data, corroborating the great potential of these hybrid scaffolds for the development of innovative devices.

## 2. Results

### 2.1. Mechanical Analysis of Chitosan-Based Sponges

**Figure 1:**
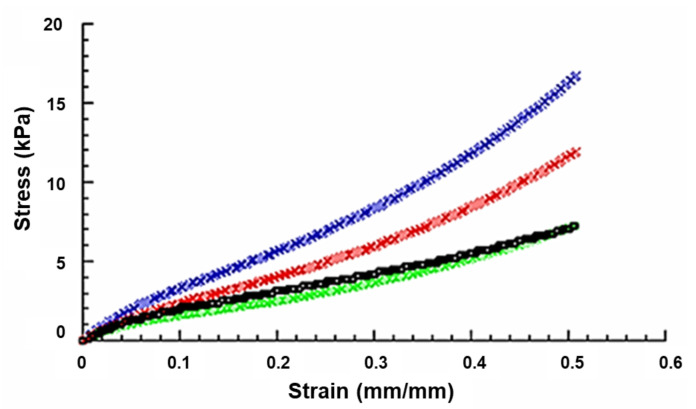
Typical stress–strain curves obtained from compression tests on ChS (Blue), ChS+EAK (Red), ChS+EAK-IKVAV (Black), and ChS+EAK-YIGSR (Green) scaffolds (rate of 1 mm/min, final strain of 50%).

Before the fabrication of the hybrid scaffolds, mechanical compression tests were performed on the four groups of chitosan-based sponges (ChS; ChS+EAK; ChS+EAK-IKVAV; ChS+EAK-YIGSR). The scaffolds showed a behaviour similar to that of a flexible foam, and the stress–strain curves are reported in Figure 1.

The compressive modulus (E) was calculated as the slope of the initial linear region of the stress–strain curve. Table 1 shows the compressive modulus (E) and maximum stress (σ_max_) as mean value ± standard deviation.

With regard to the statistical analysis, the *p*-value corresponding to the F-statistic of ANOVA was much lower than 0.05 (and 0.01), suggesting that one or more pairs of treatments were significantly different. Thus, Tukey’s HSD post hoc test was used to identify which of the pairs of treatments were significantly different from each other (Table 2).

The highest values of compressive modulus and maximum stress were observed for peptide-free ChS (Table 1 and Table 2).

The compressive modulus and the maximum stress significantly decreased from 40.4 ± 3.1 kPa to 31.1 ± 2.1 kPa and from 16.8 ± 1.0 kPa to 11.9 ± 0.6 kPa, respectively, due to the inclusion of the EAK to ChS (Table 1 and Table 2). Furthermore, if compared to ChS, a significant decrease in compressive modulus and maximum stress also occurred in the presence of the adhesive sequences IKVAV and YIGSR bonded to EAK (Table 1 and Table 2). In particular, ChS+EAK-IKVAV and ChS+EAK-YIGSR showed the values of a compressive modulus (30.9 ± 1.9 kPa and 30.6 ± 2.0 kPa, respectively) and maximum stress (7.3 ± 0.4 kPa and 7.2 ± 0.4 kPa, respectively), which were significantly lower than those observed for ChS (Table 1 and Table 2). However, as regards compressive modulus and maximum stress, no statistically significant differences were found between ChS+EAK-IKVAV and ChS+EAK-YIGSR (Table 1 and Table 2).

Furthermore, with regard to +EAK groups, even though the maximum stress found in the case of ChS+EAK (11.9 ± 0.6 kPa) was significantly higher than that obtained for ChS+EAK-IKVAV (7.3 ± 0.4 kPa) and ChS+EAK-YIGSR (7.2 ± 0.4 kPa), in terms of compressive modulus no statistically significant differences were observed among these three groups (Table 1 and Table 2). Accordingly, the adhesive sequences IKVAV and YIGSR bonded to EAK negatively affected the maximum stress of ChS+EAK without significantly altering the compressive modulus.

### 2.2. Fabrication and Morphology of OxPVA/ChS Hybrid Scaffolds

A weighted amount of chitosan, dissolved in acetic acid solution without/with the adhesive sequences (+EAK; +EAK-IKVAV; +EAK-YIGSR) was prepared to fabricate the sponges by freeze-drying and transferring to moulds (24-well plate).

Lyophilized chitosan-based sponges resulted in discoidal, white matrices with a diameter of 1.5 cm, compatible with the dimension of the mould used for their fabrication. Macroscopically, the ChS+EAK-IKVAV showed a less tight network than that of the other experimental groups, in accordance with the investigated mechanical properties (see Section 2.1).

**Figure 2:**
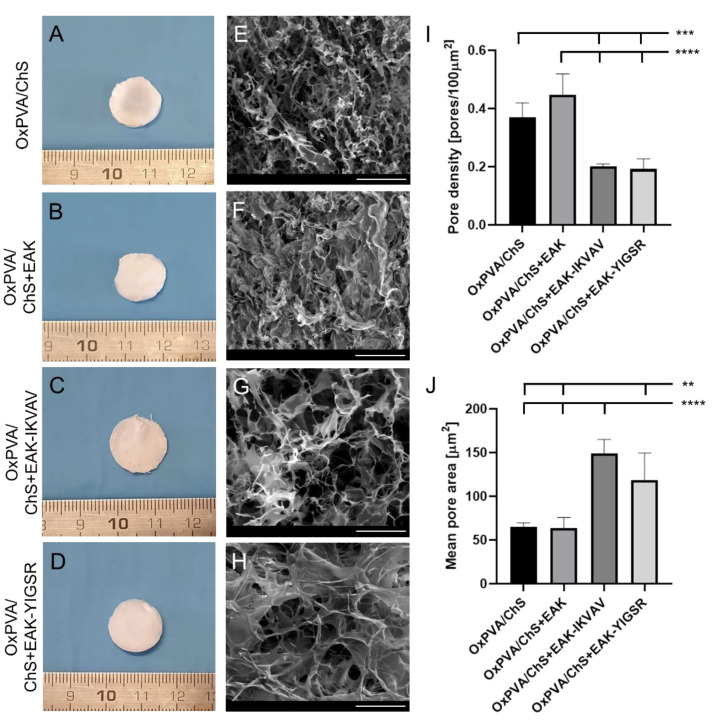
Macroscopic and ultrastructural features of OxPVA/ChS-based scaffolds. (**A**–**D**) Gross appearance of hybrid scaffolds’ seeding surfaces showing the chitosan-based layer of the supports, without/with biochemical cues (+EAK, +EAK-IKVAV, +EAK-YIGSR). (**E**–**H**) Ultrastructural characteristics of the ChS investigated by Scanning Electron Microscopy; a different fine pores interconnection is identifiable, according to the specific functionalization sequences. Scale bar: 100µm (**E**–**H**). (**I**) Pore density, calculated as total pores/100 µm^2^ area (*** *p* < 0.001; **** *p* < 0.0001) and (**J**) mean pore area [µm^2^] (** *p* < 0.01; **** *p* < 0.0001), by ImageJ software.

After fabrication, the sponges were carefully positioned over the OxPVA polymer solution and the physical cross-linking occurred by six freezing-thawing (FT) cycles (−80 °C for 1 h/RT for 1 h), thus leading to the stable coupling of the two layers (Figure 2A–D). The protocol for the scaffolds’ development preserved sponges’ integrity: OxPVA solution did not impregnate and/or cover the chitosan seeding surfaces. Additionally, the synthetic polymer was guaranteed for the high manipulability of the chitosan matrices.

The surface ultrastructure of the OxPVA/ChS hybrid scaffolds was examined by Scanning Electron Microscopy (SEM) (Figure 2E–H), confirming the sponge-like macroscopic appearance of the chitosan layers. Moreover, further details on fine chitosan polymer organization were also inferable: microporous interconnections were tighter in both the OxPVA/ChS and the OxPVA/ChS+EAK groups than in the OxPVA/ChS+EAK-IKVAV or OxPVA/ChS+EAK-YIGSR groups. As showed in Figure 2I,J, ImageJ software-based analysis corroborated that. Higher pore density was detected in OxPVA/ChS+EAK (0.45 ± 0.07) and OxPVA/ChS (0.37 ± 0.05) vs. OxPVA/ChS+EAK-IKVAV (0.20 ± 0.01) and OxPVA/ChS+EAK-YIGSR (0.19 ± 0.03) with significant differences comparing OxPVA/ChS vs. OxPVA/ChS+EAK-IKVAV and OxPVA/ChS+EAK-YIGSR (*p* < 0.001) and OxPVA/ChS+EAK vs. ChS+EAK-IKVAV and OxPVA/ChS+EAK-YIGSR (*p* < 0.0001). Considering the mean pore area, larger pores were found into OxPVA/ChS+EAK-IKVAV (149.30 ± 15.77) and OxPVA/ChS+EAK-YIGSR (118.71 ± 30.83) vs. OxPVA/ChS (64.90 ± 4.80) and OxPVA/ChS+EAK (63.89 ± 12.0). Significant differences were observed between: OxPVA/ChS+EAK-YIGSR vs. OxPVA/ChS and OxPVA/ChS+EAK (*p* < 0.01) and OxPVA/ChS+EAK-IKVAV vs. OxPVA/ChS and OxPVA/ChS+EAK (*p* < 0.0001).

### 2.3. Bioactive Potential of the Hybrid Scaffolds

The enhanced bioactivity of the OxPVA/chitosan scaffolds was assessed by testing their interaction with SH-SY5Y cells. After cell seeding on the hybrid scaffolds, the MTT assay was performed at 3 and 7 days to evaluate cell viability and growth on the supports. Hence, eventual differences in cells adhesion/proliferation induced by ChS bioactivation (+EAK, +EAK-IKVAV, or +EAK-YIGSR) were verified vs. the peptide-free group.

**Figure 3:**
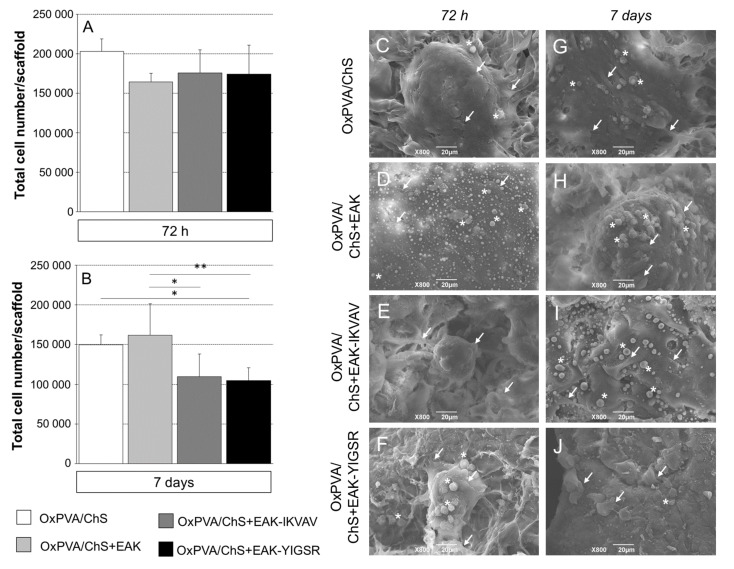
(**A**,**B**) SH-SY5Y cell adhesion and proliferation on different OxPVA/ChS-based scaffolds, evaluated by MTT assay. Bioactivation occurred through the incorporation of biochemical stimuli (+EAK, +EAK-IKVAV, and +EAK-YIGSR) within the chitosan layer. (**A**) At 72 h from seeding, no statistically significant difference in cell adhesion was observed comparing groups. Differences in proliferation were detected since day 7. (**B**) The higher total cells number was displayed by OxPVA/ChS+EAK scaffolds, while the lower cells number was observed on OxPVA/ChS+EAK-YIGSR scaffolds (* *p* < 0.05; ** *p* < 0.01). (**C**–**J**) OxPVA/ChS-based scaffolds surface analysed by SEM at 72 h (**C**–**F**) and 7 days (**G**–**J**) from SH-SY5Y cell seeding. Specifically, the experimental groups included: OxPVA/ChS (**C**,**G**); OxPVA/ChS+EAK (**D**,**H**); OxPVA/ChS+EAK-IKVAV (**E**,**I**); and OxPVA/ChS+EAK-YIGSR scaffolds (**F**,**J**). Scaffolds surface ultrastructure was not distinguishable once colonized by cells (white arrows). Representative clumps with roundish cells are shown (white asterisk). Scale bar: 20 μm.

As showed in Figure 3A,B, the chitosan-based layers without/with biochemical cues were all able to induce cell adhesion and proliferation over OxPVA supports. This is interesting evidence; in fact, as broadly demonstrated, smooth OxPVA scaffolds did not trigger cell adhesion and proliferation in vitro [10,11,14]. Initially, at 72 h from seeding, a certain growth trend was detected in the whole cohort, but no specific differences were identifiable when comparing the groups (Figure 3A). The safeness of the scaffolds’ preparation phases was also corroborated by these data. At day 7 from seeding, some statistical differences emerged (Figure 3B). Considering the proliferation data, the higher total cells number was observed on OxPVA/ChS+EAK (*p* < 0.05 vs. OxPVA/ChS+EAK-IKVAV; *p* < 0.01 vs. OxPVA/ChS+EAK-YIGSR) followed by OxPVA/ChS (*p* < 0.05 vs. OxPVA/ChS+EAK-YIGSR).

### 2.4. SEM Analysis of Cells Distribution on OxPVA/Chitosan-Based Scaffolds

To qualitatively support the MTT assay results, the presence of SH-SY5Y cells on OxPVA/Chitosan-based supports was also investigated by SEM at 72 h and 7 days from seeding (Figure 3C–J). The samples, properly fixed (2.5% glutaraldehyde in a 0.1 M cacodylate buffer) and dehydrated (graded ethanol series) allowed for the analysis.

Adherent cells were observed in the whole cohort at each endpoint, confirming the MTT data. Focusing on cell organization over the scaffolds’ surfaces, colonies were detected after 72 h from seeding. After 7 days, thicker clusters of cells were visible. Extensive neurite outgrowths, suggesting differentiation into neuronal-like cells, were not observed; conversely, as a consequence of massive proliferation, cells tended to form clumps appearing as overlapping cells, growing on top of one another in the central region of a cell mass. Cells appeared to acquire a roundish morphology up to detachment (see Figure 3C,D,F,J).

### 2.5. In Vivo Biocompatibility of OxPVA/Chitosan-Based Hybrid Scaffolds

The biocompatibility of the hybrid scaffolds was assessed by an in vivo implant into the subcutaneous region of BALB/c mice in order to evaluate the immunological and foreign-body reactions, as well as the fibrous encapsulation of the implanted biomaterials.

**Figure 4:**
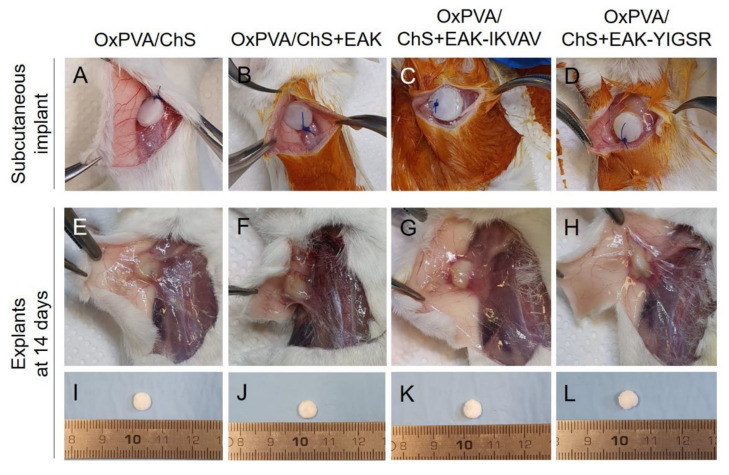
Gross appearance of OxPVA/ChS-based scaffolds grafted into the subcutaneous tissue of BALB/c mice (**A**–**D**) and 14 days from surgery, at the implant site (**E**–**H**) and after sample excision (**I**–**L**).

When anchored to the *latissimus dorsi* muscle of the animals, the four types of scaffolds exhibited adequate suture retention capacity (Figure 4A–D). At the time of sample retrieval, 14 days from surgery (Figure 4E–L), all scaffolds were well identifiable at the implant site and no macroscopic signs of inflammation were visible within the tissues surrounding the graft. A thin connective capsule enveloping the scaffolds was macroscopically recognizable, compatible with grade 1 soft tissue adhesion.

**Figure 5:**
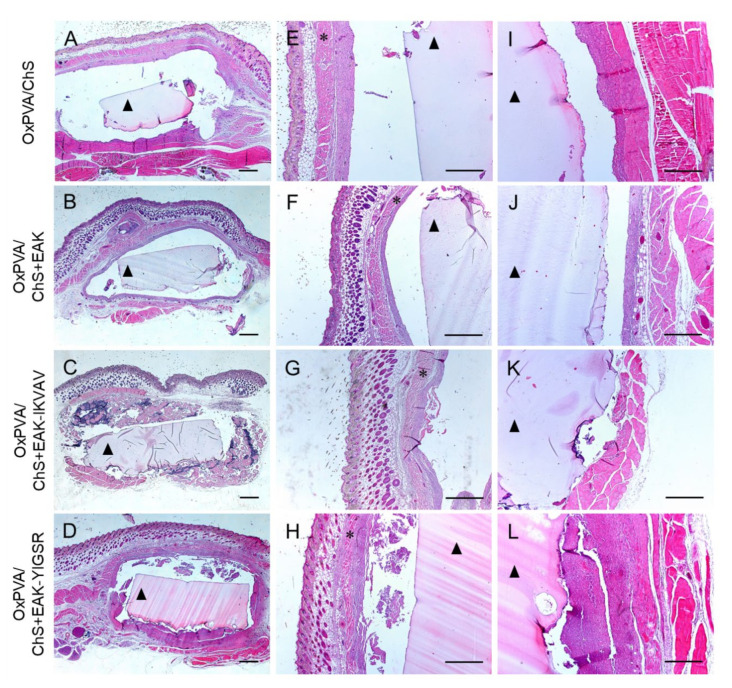
Hematoxylin and Eosin staining of OxPVA/ChS-based scaffolds integrated with surrounding host tissues at the site of implant (**A**–**D**) and preliminary evaluation of the tissues at the superficial (**E**–**H**) and deep (**I**–**L**) aspects of the grafts. Scale bar: 800 µm (**A**–**H**); 400 µm (**I**–**L**). (*: the *panniculus carnosus* within the host subcutaneous tissue; ^▲^: the OxPVA-based implants).

Explanted samples were properly fixed in formalin solution and paraffin-embedded and processed for subsequent histological and immunohistochemical analyses. The histological investigation by haematoxylin and eosin (H&E) staining allowed to preliminarily evaluate the graft and the surrounding host tissues then analysed for their typical microscopic morphology (Figure 5A–D). At the superficial aspect of the implants, the multiple skin tissue layers were appreciable in all samples, including epidermis, dermis, white adipose tissue, and the *panniculus carnosus* (i.e., the thin striated muscle layer, localized between the adipose tissue and the interstitial connective tissue) (Figure 5E–H). At the deep aspect of the implants, the *latissimus dorsi* muscle was visible, which served for scaffold anchorage with the chitosan layer in contact with the muscular tissue (Figure 5I–L). At the microscopic analysis, a fibrous reactive capsule was confirmed to be completely surrounding the implant in all the experimental groups, being consistent with a normal host reaction towards biomaterial implantation. No evident differences were observed regarding this reactive capsule at the subcutaneous side vs. the *latissimus dorsi* muscle layer.

**Figure 6:**
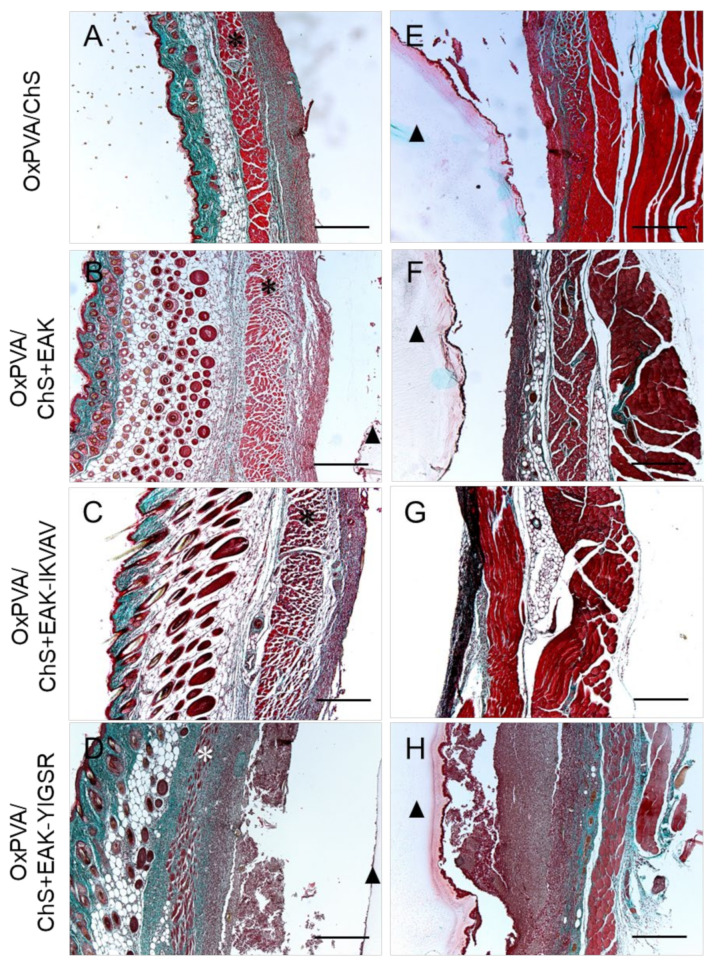
Masson’s Trichrome staining of subcutaneously implanted OxPVA/ChS-based scaffolds for the evaluation of the fibroconnective tissues (collagen, green) enveloping the superficial (**A**–**D**) and deep (**E**–**H**) aspects of the grafts. Scale bar: 400 µm. (*: the *panniculus carnosus* within the host subcutaneous tissue; ^▲^: the OxPVA-based implants).

In parallel, Masson’s Trichrome staining corroborated the presence of a moderate fibrotic reaction towards the hybrid scaffolds, as indicated by the fibroconnective capsule composed of dense, compacted collagen (green colour), which surrounds all the grafts at the interface with the host tissue at both the superficial (Figure 6A–D) and deep (Figure 6E–H) aspects of the implants.

**Figure 7:**
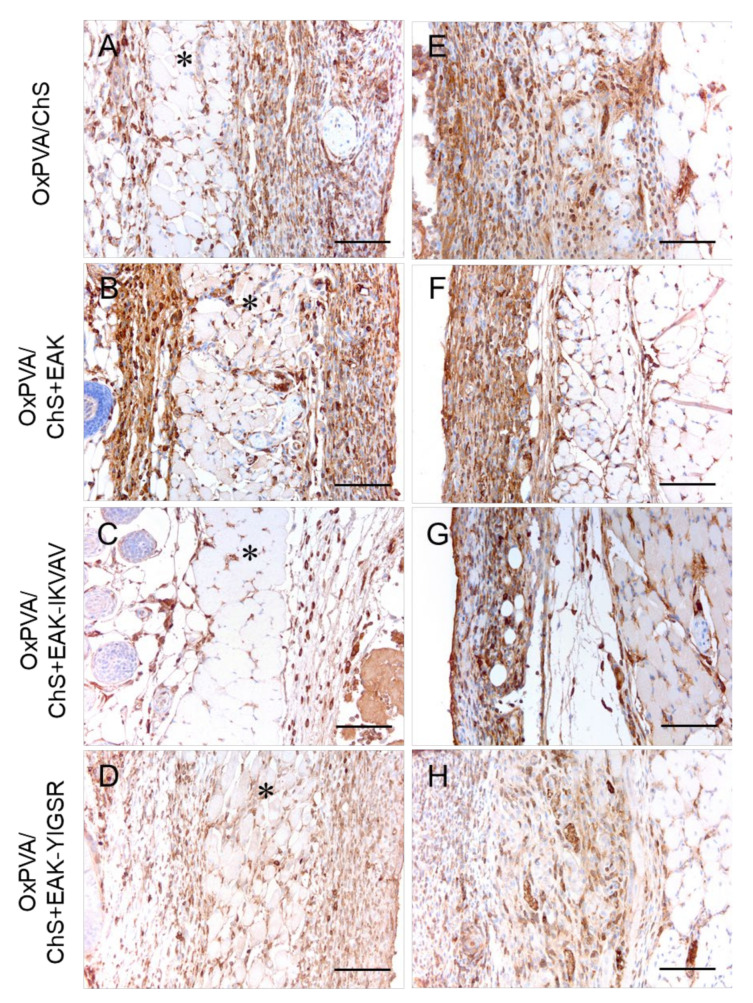
Immunolocalization of the lymphocyte T (CD3+ cells) infiltration at the superficial (**A**–**D**) and deep (**E**–**H**) aspects of the grafts after subcutaneous implantation of OxPVA/ChS-based scaffolds for 14 days. Scale bar: 100 µm. (*: the *panniculus carnosus* within the host subcutaneous tissue).

**Figure 8:**
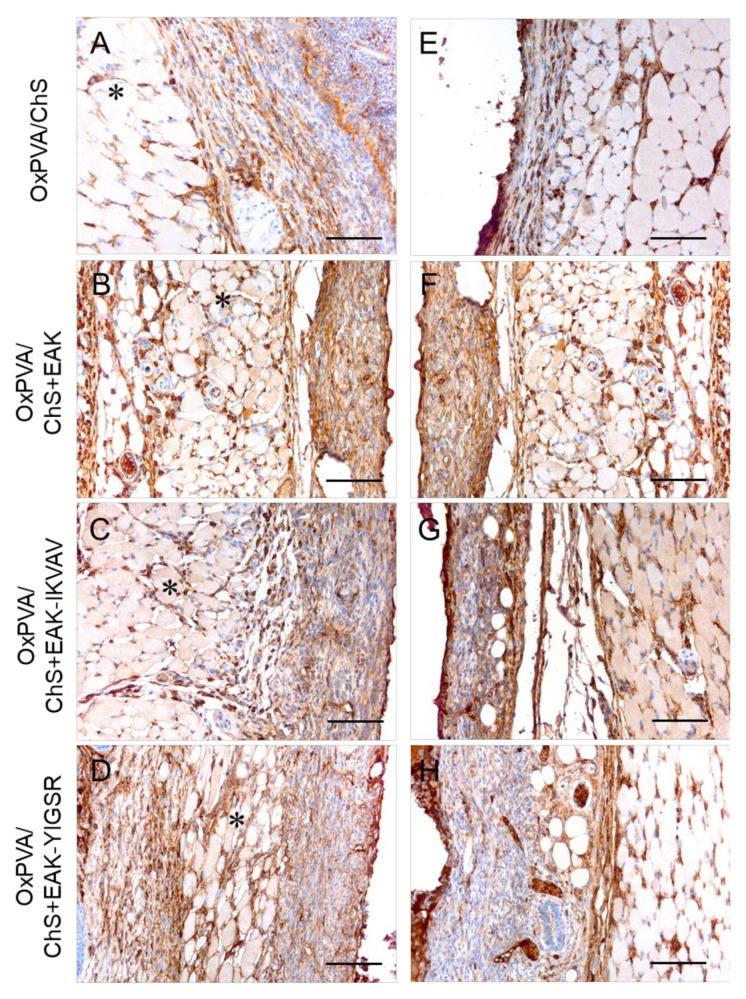
Immunolocalization of the monocyte-macrophage (F4/80+ cells) infiltration at the superficial (**A**–**D**) and deep (**E**–**H**) aspects of the grafts after the subcutaneous implantation of OxPVA/ChS-based scaffolds for 14 days. Scale bar: 100 µm. (*: the *panniculus carnosus* within the host subcutaneous tissue).

The immune response towards the grafted hybrid scaffolds was better characterized by the immunolocalization of lymphocytes T (CD3+) (Figure 7) and monocytes/macrophages (F4/80+) (Figure 8) cells at the host–graft interface. The immunohistochemical analysis showed that a moderate lympho/monocyte infiltration was triggered by all the scaffolds, with CD3- and F4/80-positive cells was mainly localized at the level of the reactive capsule delimiting the hybrid scaffolds at both the superficial (Figure 7A–D and Figure 8A–D) and deep (Figure 7E–H and Figure 8E–H) aspects of the implant. A few positive cells were also immunolocalized within the subcutaneous structures and deeper muscular layer.

**Figure 9:**
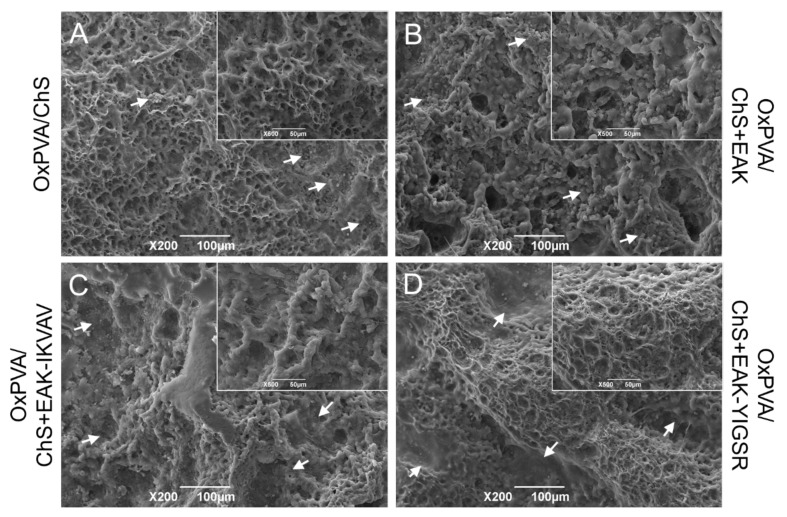
(**A**–**D**) SEM investigation of OxPVA/ChS-based scaffold surface ultrastructure after subcutaneous implantation into BALB/c mice for 14 days. Host cells were observed to adhere to the chitosan layers of all hybrid scaffolds (white arrows). Scale bar: 100 µm. Higher magnification details for each group are shown in the inserts. Scale bar: 50 µm.

Finally, ultrastructural analysis by SEM allowed the appreciation of the microporous aspects of the chitosan-based sponges cross-linked on the surface of OxPVA hydrogels (Figure 9). Due to exposure to the physiological environment, a slight modification of the scaffolds surface ultrastructure was detected, compared to the pre-implant appearance; however, a certain fine porosity was still recognizable. Remarkably, host cells were observed to adhere on the chitosan layer of all the hybrid supports, confirming their adhesive properties also in the in vivo setting.

## 3. Discussion

In case of severe PNI, guaranteeing a satisfactory nerve regeneration still represents an unmet clinical need, thus revealing as an intriguing challenge still facing the tissue engineering field. In this context, research on new biomaterials and/or biomaterial combinations is particularly fervent, aiming to fabricate a NC able to support the adhesion, proliferation, and migration of nerve and glial cells. In particular, combining natural and synthetic polymers for a synergistic effect appears highly appealing, allowing the optimization of the advantages of both in terms of mechanical properties and biological functions [21]. This strategy was attempted here: within this study, discoidal hybrid scaffolds based on differently functionalized chitosan sponges + the new polymer OxPVA were developed and characterized both in vitro/in vivo, guiding toward future hybrid tubular devices.

Chitosan is a polysaccharide broadly used for supporting peripheral nerve regeneration, fulfilling the recommendations for a nerve conduit material [22]. In fact, chitosan-based devices show adequate biodegradability, biocompatibility, neglectable toxicity, and suitable biological absorptivity; excellent antibacterial activity is also reported [22,23,24,25]. In addition, chitosan proved beneficial in guiding Schwann cells’ orientation, preserving the survival and differentiation of neuronal cells (commercial device, Reaxon^®^). All this evidence highlights its potential to serve as a valid alternative to autografts [22,24,25]. However, although distinguished as a highly promising material for NCs, positive outcomes are mainly related to short gaps, whereas its contribution in long gap nerve injury repair still needs to be clarified due to conflicting results [22,26]. To predict the in vivo crosstalk of the supports considered here, the human neuroblastoma cell line SH-SY5Y was used for in vitro studies, as these cells share certain properties with primary neurons [17].

To date, different modifications have been reported in the literature, proving the necessity of intense research to enhance chitosan in vivo performances. These include grafting with bioactive peptides, the alteration of the surface ultrastructure and combination with other polymers [27]. Within this study, the aim was to guide future research in NCs development but also in tissue engineering approaches, based on chitosan.

Cell-adhesive molecules derived from ECM (collagen, fibronectin, vitronectin, and laminin) can be effectively adsorbed on chitosan substrates [28,29,30]. However, despite encouraging evidence, easy desorption is likely to occur, prompting research into chitosan chemistry for more stable alternatives [29]. Surface chemical modification with immobilized, bioactive peptides likely assures a higher control and selectivity over cell–biomaterial interactions than other strategies [31]. As reviewed by Hozumi and Nomizu [32], several reagents/protocols have been reported for binding peptides to chitosan, intended for different destinations. Among these, 1-ethyl-3-(3-dimethylaminopropyl)-carbodiimide and N-hydroxy succinimide (NHS) were adopted for the immobilization of RGDS (Arg-Gly-Asp-Ser) carboxyl residue to the amine groups of chitosan (end use: bone) [33]. NHS and suberic acid bis (N-hydroxy-succinimide ester) were used for chitosan amine end group activation and conjugation with the amino terminal of the RGD sequence or epidermal growth factor (EGF) (end use: cartilage) [34]. Masuko et al. [35] introduced 2-iminothiolane to the chitosan amine residue and immobilized the cysteine-containing peptide through the disulfide bond formation (end use: synthetic ECM development). Hozumi and Nomizu [36] introduced the N-(m-maleimidobenzoyloxy) succinimide to the amine residue of chitosan, and then the cysteine-containing peptides were immobilized in the maleimidobenzoyl group through the mercapto group (end use: synthetic ECM development). Interestingly, in order to specifically modify neural cell adhesion to chitosan-based substrates and support neurite outgrowth, the bioactive laminin sequences YIGSR (eventually, with a glycine spacer, Wang et al. [37] bonded IKVAV to thiolated chitosan, forming chitosan-S-S-laminin peptide structures [29,38]. All these literature-based examples clearly highlight that chitosan is an excellent platform for tissue engineering purposes and is highly prone to functionalization due to its high content in various hydroxyl and amine groups [39]. However, utilizing chemical agents to stably decorate chitosan supports implies possible undesired effects connected to toxic remnants entrapment. To bypass this possibility, a different strategy was pursued here, examining SAPs dispersion in chitosan; furthermore, a focus over specifically bioactive sequences for neural regeneration was maintained because of IKVAV and YIGSR sequence inclusion.

The SAPs are protein fragments that in solution self-organize in very stable amphiphilic-beta sheets conformation by hydrogen bonds; thus, they have great potential in tissue engineering, including peripheral nerve regeneration [20,40,41] due to a 3D self-arrangement mimicking ECM ultrastructural organization [41,42]. Furthermore, they also display high stability against thermal, chemical, and proteolytic attacks [43]. Avoiding adsorption (less stable) and covalent binding (possible chemical residues entrapment), the SAP EAK was here dissolved into a chitosan solution obtaining a complex ECM-like ultrastructure stabilized by secondary bonds; a similar approach was recently reported, for the first time in the literature, in order to enrich OxPVA hydrogels [17]. Interestingly, as SAPs do not have specific motifs promoting cell adhesion/growth likely associated with the nanometric fibrous scaffold or intrinsic mechanical properties [42], IKVAV (promotes adhesion/differentiation/neurons’ axons growth/stimulates adequate microenvironment) or YIGSR (promotes cell adhesion) was grafted to EAK. The development of conjugates is expected to sustain the chitosan sponges’ bio-effect [44,45].

Together with a favourable chemistry allowing bioactivation, chitosan also displays high mouldability, allowing for the fabrication of different scaffolds according to end-use destination (e.g., nanoparticles, hydrogels) [46]; here, sponge fabrication was chosen. In addition, the combination with other materials can easily occur. Specifically, regarding hybridization with polymers, the literature reports about several hybrids and/or composites combining the chitosan advantages with that, for instance, of collagen, polyglycolic acid (PGA), polylactic acid (PLA), poly(Lactic-Co-glycolic Acid) (PLGA), and polyethylene glycol [27]. The hybrid approach mainly revealed the superior results of axonal recovery compared to chitosan alone. OxPVA, a new material with an established promising attitude in supporting peripheral nerve regeneration [14,15,16,17,47], was here coupled with the ChS. The aim of this coupling was to improve OxPVA biological behaviour; in fact, as broadly reported, cell adherence was inhibited by the highly hydrophilic nature of the OxPVA hydrogel [14,17]. In the past, hybridization (SAPs, ECM derivatives) [9,10,17] and/or ultrastructural modification (patterning) [17] was revealed to be fundamental in triggering cell/biomaterial interactions in vitro.

As it is possible to achieve OxPVA-based scaffolds through physical crosslinking, OxPVA/ChS, OxPVA/ChS+EAK, OxPVA/ChS+EAK-YIGSR, and OxPVA/ChS+EAK-IKVAV supports were obtained through a completely “safe” strategy (freeze-thawing) not involving chemical agents (likely highly adsorbed due to a certain hydrogel swelling) and assuring the maintenance of OxPVA specific features (e.g., mechanical characteristics, permeability, biodegradation rate). In accordance with previous in vitro characterization studies [12,13,16] and preclinical evidence [14,15], OxPVA has mechanical characteristics that well match the requirements of the surgeon (no ruptures during NCs grafting), end-use destination (no NCs translocations), and implant site (no NCs kinking). Additionally, good in vivo results suggest that OxPVA-based devices display an adequate permeability, which supports the establishment of a favourable physico-chemical/biological environment during nerve regeneration. Possibly, this balance could be affected if a mixed hybrid scaffold OxPVA/ChS were to be prepared, requiring intense research for the identification of the optimal volume ratio. This is, for instance, seen in the study by Xie et al. [48], considering PLA/chitosan NCs fabrication, or in the research by Nawrotek et al. [49], fabricating chitosan-hydroxyapatite + collagen and/or hyaluronic acid grafts.

The mechanical properties of the chitosan-based sponges were investigated in order to appreciate eventual differences likely amenable to the presence and/or type of bioactive cues. Experimental data show a difference among the samples, highlighting a reduction in compressive modulus and maximum stress along with both a presence of SAPs and chain length. As regards the enriched chitosan matrices, EAK-IKVAV and EAK-YIGSR provided a contribution in reducing mechanical strength, the results of which were significantly lower than those of matrices with only EAK, without negatively affecting the compressive modulus. Despite this, these values do not affect the overall OxPVA/ChS-based scaffolds’ mechanical properties (mainly dictated by the OxPVA polymer [9,12,13]), and they may correlate with ChS ultrastructure and crosstalk with cells and thus influence cell behaviour after seeding.

A preliminary consideration may associate the mechanical properties of the sponges with their ultrastructure. Lower mechanical properties likely imply a less tight organization of the ChS-based matrices; accordingly, larger mesh networks were evident in ChS+EAK-IKVAV and ChS+EAK-YIGSR (however, comparable among them) rather than in ChS+EAK and ChS alone; that being said, with regard to ultrastructure, image analyses also provided further information (higher pore density in ChS+EAK and ChS alone than ChS+EAK-IKVAV/-YIGSR; higher pore area in ChS+EAK-IKVAV/-YIGSR than ChS and ChS+EAK). Possibly, this may be ascribed to different chains steric hindrance, leading to a distinct spatial arrangement. In turn, the lower modulus of ChS+EAK-IKVAV and ChS+EAK-YIGSR (which was, however, not significantly different from that of ChS+EAK) may also correlate with cells penetration within the sponges, which was generally favoured [42]. Thus, the synergistic combination of a larger mesh network with a lower modulus may play a crucial role in cell behaviour. Overall, the obtained results showed the potential of tailoring the mechanical properties of the ChS-based scaffolds according to the specific application.

After seeding (72 h), all the matrices showed promising behaviour in supporting interaction with SH-SY5Y cells which, in turn, displayed a typical cluster-like organization [50]. At day 7, these clumps-like growths resulted in a certain number of cell reductions because of their possible detach due to overlapping and no surface available for adhesion. It is likely that, despite the ultrastructural differences discussed, all the chitosan-based networks were complex enough to induce cell adhesion and proliferation [50]. The ability to support interaction with cells is an interesting achievement, especially when considering that chitosan alone has no cell attachment activity, thus requiring functionalization/surface modification, in turn behaving like a modifiable platform [32]. Regarding this, an in vitro study by Luna et al. [51] is enlightening. The authors demonstrated that, whether untreated or treated (by argon or nitrogen-plasma), smooth chitosan membranes do not induce either cell adherence nor proliferation with increasing culturing times. This reveals that chitosan ultrastructural modification is an essential prerequisite to confer a certain bioactivity to chitosan and trigger its interaction with cells. Furthermore, as SAPs hydrogels can be designed to affect the network elasticity (specific peptide length, bioactive motifs), they can be exploited as a physiochemical regulator of cellular fate. As occurs in vivo, integrin-binding mediated events communicate the mechanics of the ECM to the cells and direct their fate through intracellular signalling pathways [52]. Within this scenario, a mechanical contribution from the OxPVA layer, above which the sponges are crosslinked, cannot be excluded.

The possibility of fabricating biocompatible scaffolds by means of bio-safe and reliable approaches is often a critical step towards the manufacture of tissue-engineered devices and their future translation into the market [53]. Biocompatibility is an important requisite for the success of a biomaterial for biomedical use, which will be in direct contact with living tissues; hence, this feature can be preliminarily assessed evaluating the intensity of an inflammatory reaction of the tissues adjacent to the implant (orthotopic implant) by histological/immunohistochemical analyses. Typically, lymphocytes and macrophage invasions are considered when examining eventual chronic inflammation and, in particular, macrophages are recognized as key mediators of immune reactions towards biomaterials [54]. According to experimental evidence, the tissues’ reaction to the hybrid OxPVA/ChS-based membranes appeared to be favourable; only moderate inflammatory infiltration and a tolerable fibrous capsule were detected, without severe foreign body reaction signs. These data are a further confirmation regarding the “safe” fabrication methods of both the two layers and their coupling. In addition, it was also possible to recognize the two coupled sheaths’ integrity (histological analysis and SEM), as well as the maintenance of the ChS-derived strata ultrastructure, confirming the adequacy of OxPVA/ChS crosslinking and suggesting sample stability not only towards the in vivo environment but also in surgery manipulation/suture for positioning.

## 4. Materials and Methods

### 4.1. Development and Analysis of Chitosan-Based Sponges

#### 4.1.1. Peptides’ Synthesis and Purification

Three different peptides were synthetized for the subsequent development of functionalized ChS. Specifically, the bioactive sequences included the SAPs EAK, EAK-IKVAV, and EAK-YIGSR. 

The Rink Amide MBHA resin and the Fmoc protected amino acids were purchased from Novabiochem (Merck KGaA, Darmstadt, Germany). TES was purchased from Sigma Aldrich (Steinheim, Germany). TFA was purchased from Biosolve (Valkenswaard, The Netherlands). Chitosan 70/1000 was purchased from Heppe Medical Chitosan GmbH (Halle, Germany). All other reagents and solvents were purchased from Novabiochem (Merck KGaA, Darmstadt, Germany).

The peptide EAK (sequence: H-Ala-Glu-Ala-Glu-Ala-Lys-Ala-Lys-Ala-Glu-Ala-Glu-Ala-Lys-Ala-Lys-NH_2_) was synthesized on Rink Amide MBHA resin (0.52 mmol/g) using Fmoc chemistry by a Syro I synthesizer (Multisyntech, Witten, Germany). The side-chain protecting groups were OtBu, Glu, and Boc, Lys. All the couplings were double (for each coupling 5 equivalents of Fmoc-amino acid; 5 eq. HBTU; 5 eq. oxyma pure; and 10 eq. DIEA were used). After the Fmoc-deprotection of the last inserted amino acid, the peptide was deblocked from the resin and deprotected from side-chain protecting groups using the mixture 4.75 mL TFA, 0.125 mL TES, and 0.125 mL H_2_O, for 1.5 h. The resin was filtered off and the solution was concentrated and added with cold diethyl ether. The product was precipitated and filtered. The identity of the crude peptide was determined by mass spectrometry (expected mass = 1614.49 Da; theoretical mass = 1614.79 Da; AB-SCIEX TOF-TOF 4800 instrument, Figure S1A). The peptide EAK was isolated by RP-HPLC and characterized by analytical RP-HPLC (conditions: Vydac C_18_ column (5 µm, 300 Å, 4.6 × 250 mm, Grace), eluent A: 0.05% TFA in H_2_O; eluent B: 0.05% TFA in CH_3_CN; gradient: from 5 to 20% di B in 30 min, flow rate: 1 mL/min; detector: 214 nm. t_R_ = 21.62 min, Figure S1B). The integration of the chromatogram confirmed a 94% purity grade.

The peptide EAK-IKVAV (sequence: H-Ala-Glu-Ala-Glu-Ala-Lys-Ala-Lys-Ala-Glu-Ala-Glu-Ala-Lys-Ala-Lys-Ile-Lys-Val-Ala-Val-NH_2_) was synthesized on Rink Amide MBHA resin (0.52 mmol/g) using Fmoc chemistry by a Syro I synthesizer (Multisyntech, Witten, Germany). The side-chain protecting groups were OtBu, Glu, and Boc, Lys. All the couplings were double. After Fmoc-deprotection, the peptide was deblocked from the resin and deprotected from side-chain protecting groups using the mixture 4.75 mL TFA, 0.125 mL TES, 0.125 mL H_2_O, for 1.5 h. The resin was filtered off and the solution was concentrated. The product was precipitated with diethyl ether and filtered. The identity of the crude peptide was determined by MALDI mass spectrometry (expected mass = 2125.73 Da; theoretical mass = 2125.48 Da; AB-SCIEX TOF-TOF 4800 instrument, Figure S2A). The peptide EAK-IKVAV was purified by RP-HPLC and characterized by analytical RP-HPLC (conditions: Nova-Pak HR C_18_ column (4 µm, 60 Å, 3.9 × 300 mm, Waters), eluent A: 0.05% TFA in H_2_O; eluent B: 0.05% TFA in CH_3_CN; gradient: from 15 to 30% di B in 30 min, flow rate: 1 mL/min; detector: 214 nm. t_R_ = 17.61 min, Figure S2B). The integration of the chromatogram gave a 98% purity grade.

The peptide EAK-YIGSR (sequence: H-Ala-Glu-Ala-Glu-Ala-Lys-Ala-Lys-Ala-Glu-Ala-Glu-Ala-Lys-Ala-Lys-Tyr-Ile-Gly-Ser-Arg-NH_2_) was synthesized on Rink Amide MBHA resin (0.52 mmol/g) using Fmoc chemistry by a Syro I synthesizer (Multisyntech, Witten, Germany). The side-chain protecting groups were: OtBu, Glu; Boc, Lys; Pbf, Arg; and tBu, Ser, and Tyr. All the couplings were double. After Fmoc-deprotection, the peptide was deblocked from the resin and deprotected from side-chain protecting groups using the mixture 4.75 mL TFA, 0.125 mL TES, and 0.125 mL H_2_O, for 1.5 h. The resin was filtered off and the solution was concentrated. The product was precipitated with diethyl ether and filtered. The identity of the crude peptide was determined by MALDI mass spectrometry (expected mass = 2192.06 Da; theoretical mass = 2191.45 Da; AB-SCIEX TOF-TOF 4800 instrument, Figure S3A). The peptide EAK-YIGSR was purified by RP-HPLC and characterized by analytical RP-HPLC (conditions: Nova-Pak HR C_18_ column (4 µm, 60 Å, 3.9 × 300 mm, Waters), eluent A: 0.05% TFA in H_2_O; eluent B: 0.05% TFA in CH_3_CN; gradient: from 18 to 26% di B in 24 min, flow rate: 1 mL/min; detector: 214 nm. t_R_ = 10.68 min, Figure S3B). The integration of the chromatogram gave a 99% purity grade.

#### 4.1.2. Set-Up of the Chitosan-Based Sponges

Peptide-free ChS were prepared by dissolving 3.15 mg of chitosan in 472 μL of acetic acid solution 0.2 M under magnetic stirring. Of the solution, 375 mg were then poured within each 24-well plate, frozen with nitrogen liquid, and freeze-dried. After the lyophilization, cleavages were performed by filling each well three times with ethanol and later three times with MilliQ water. Finally, the scaffolds were once again frozen with nitrogen liquid and freeze-dried.

Regarding the development of the functionalized ChS, each scaffold was prepared by dissolving under magnetic stirring 0.945 mg of peptide (30% *w*/*w* peptide/chitosan), and 3.15 mg of chitosan in 472 μL of acetic acid solution 0.2 M. Of the solution, 375 mg were then poured within each 24-well plate, frozen with nitrogen liquid, and freeze-dried. After the lyophilization, cleavages were performed by filling each well three times with ethanol and later three times with MilliQ water. Finally, the scaffolds were once again frozen with nitrogen liquid and freeze-dried.

#### 4.1.3. Mechanical Analysis of the Chitosan-Based Sponges

Compression tests were performed on peptide-free ChS and functionalized ChS (radius Ro = 6 mm, height Ho = 0.7 mm). Samples were immersed in physiological solution at 37 °C and tested using an INSTRON 5566 testing machine at a rate of 1 mm/min up to a strain of 50%. Considering the measured force F, the initial cross-sectional area of the specimen (A_0_ =π R_0_^2^), the height variation (ΔH), the initial height (H_0_) of the specimen, the engineering stress (σ), and the engineering strain (ε) were calculated as follows: σ = F/A_0_ and ε = ΔH/H_0_. ANOVA and Tukey’s HSD test were used for statistical analysis (*p* < 0.05).

### 4.2. Fabrication and Analysis of the Hybrid Scaffolds

#### 4.2.1. OxPVA Solution Preparation

OxPVA solution was prepared in accordance with a protocol previously reported in Stocco et al. [8,11]. Preliminarily, a suspension of PVA powder (molecular weight (Mw) 146,000–186,000 Da, 99+% hydrolysed) (i.e., 10 g in MilliQ water) was heated in boiling bath under stirring to promote complete polymer solubilization. Thereafter, the solution was cooled at 37 °C before proceeding with partial oxidation and dialysis. Partial oxidation was performed by the addition to the PVA solution of 151 mg potassium permanganate (KMnO_4_) in 10 mL of acidic MilliQ water (1.60 g of 70% HClO_4_ (*w*/*w*)). The end of the oxidation phase corresponded to the complete discoloration of the system, which generally took 1 h at 37 °C. Hence, extensive dialysis occurred by means of 8000 Da cut-off membrane (Sigma-Aldrich). The OxPVA solution was then frozen at −20 °C overnight and lyophilized (Speed Vac Concentrator Savant, Instruments Inc., Farmingdale, NJ, USA) for long-term storage. For polymer recovering, 16 wt% OxPVA was weighted, suspended into MilliQ water, and then heated for 48 h at 100 °C.

#### 4.2.2. Hybrid Scaffolds Set-Up

**Figure 10:**
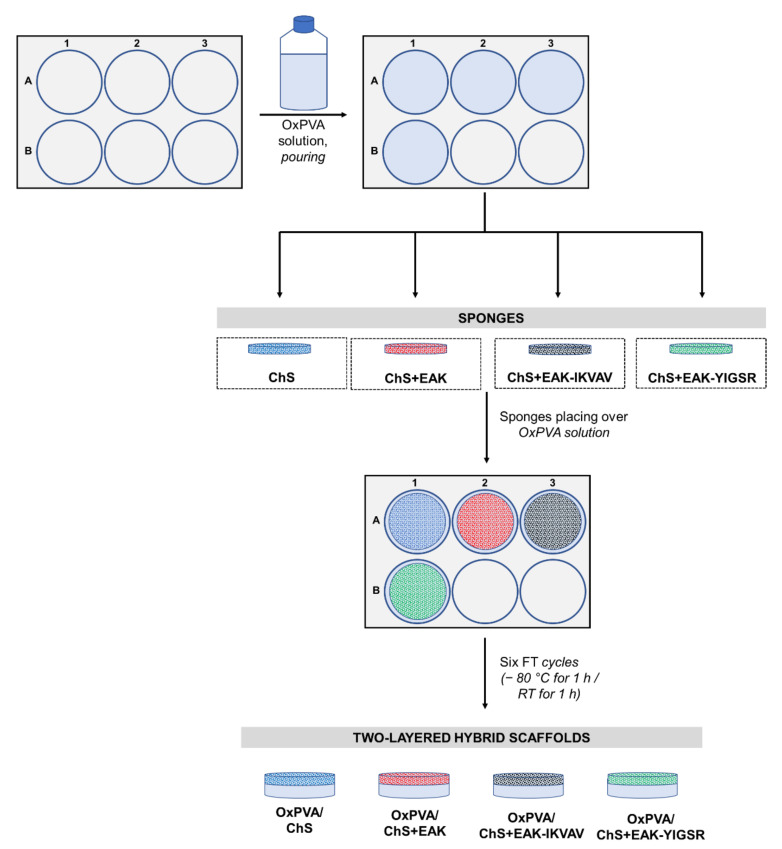
Fabrication of two-layered hybrid scaffolds based on oxidized polyvinyl alcohol (OxPVA) and bioactivated chitosan sponges. The OxPVA solution was poured into moulds; hence, the sponges comprising chitosan alone (ChS); chitosan functionalized with the self-assembling-peptide EAK (ChS+EAK); chitosan functionalized with the self-assembling-peptide EAK + the laminin-derived sequence -IKVAV (ChS+EAK-IKVAV); and chitosan functionalized with the self-assembling-peptide EAK + the laminin-derived sequence -YIGSR (ChS+EAK-YIGSR) were carefully positioned over the polymer solution. After exposing the system to six freezing thawing (FT) cycles (one cycle: −80 °C for 1 h followed by room temperature (RT) for 1 h), the two-layered hybrid scaffolds (OxPVA/ChS; OxPVA/ChS+EAK; OxPVA/ChS+EAK-IKVAV; OxPVA/ChS+EAK-YIGSR) were obtained.

Hybrid scaffolds based on OxPVA/ChS were prepared by pouring an OxPVA solution within a 6-well plate (mould), assuring the formation of a thin polymer layer (2 mm in thickness). Hence, the lyophilized chitosan matrices +/− peptides (i.e., EAK, EAK- IKVAV, EAK-YIGSR), prepared as previously described, were carefully placed over the polymer film, and a freezing–thawing (FT) treatment was adopted to physically cross-link the hydrogel and to embed the lyophilized matrix upon it. In particular, 6 FT cycles (−80 °C for 1 h/RT for 1 h) were performed, and then the two-layered hybrid scaffolds were kept at 20 °C until use (Figure 10).

#### 4.2.3. Scaffolds Ultrastructure

The ultrastructure of the OxPVA/ChS scaffolds seeding surface was investigated by SEM. After fabrication, the scaffolds were fixed with 2.5% glutaraldehyde (Sigma-Aldrich) in a 0.1 M cacodylate buffer (Sigma-Aldrich) (pH = 7.2) for 24 h, and then dehydrated through a graded ethanol series (Vetrotecnica, Padova, Italy). After critical point drying and gold sputtering, observation occurred using a Stereoscan-205 S (Cambridge Instruments, Pine Brook, NJ, USA).

Image recognition tools (ImageJ, National Institutes of Health) were utilized to investigate the surface morphology in accordance with the literature [55,56]; five photomicrographs/sample were used to calculate pores density, referred to an area of 100 µm^2^ ([number of counted pores/photomicrograph area] × 100) and mean pore area [µm^2^].

### 4.3. Bioactivity of the Hybrid Scaffolds: SH-SY5Y Cell Seeding and Proliferation Assessment

The bioactive behaviour of hybrid scaffolds was verified considering the interplay with SHSY-5Y cells by the 3-(4,5-dimethylthiazol-2-yl)-2,5-dimethyltetrazoliumbromide (MTT) assay. Hence, cell distribution over the supports was evaluated by SEM.

The hybrid scaffolds were preliminarily disinfected using a 2% antibiotic/antimycotic solution (penicillin/streptomycin, Life Technologies, Paisley, United Kingdom) for 1 h under UV light (30 min/side) and then they were washed in a DMEM/F-12 (1:1) basal medium (Life Technologies). Hence, the supports were located in 48-well plate and seeded with SH-SY5Y cells (10 × 10^4^ cells/cm^2^; European Collection of Cell Cultures, Porton Down, UK) cultured in a proliferative medium consisting of DMEM/F-12, added with 15% FBS (Fetal Bovine Serum, Sigma-Aldrich), 1% non-essential amino acids (Sigma-Aldrich) and 1% antibiotic solution; the medium was refreshed every 2 days. Cell proliferation was evaluated by MTT (0.5 mg/mL) at 72 h and 7 d from seeding; briefly, after scaffolds incubation in MTT solution (4 h at 37 °C), formazan precipitates were dissolved in 2-propanol acid (0.04 M HCl in 2-propanol) and a microplate autoreader EL 13 (BIO-TEK Instruments, Winooski, VT, USA) was adopted to measure the solution optical density at 570 nm. As previously described [57], the results of cell seeding were expressed as total cell number/scaffold, according to a MTT standard curve, preliminary prepared. To this purpose, 1000, 5000, 10,000, 20,000, and 100,000 SH-SY5Y cells/well were seeded in 96-well plates and left to adhere for 12 h. Thereafter, cell viability was measured by MTT assay, obtaining optical density values to associate to each point of the curve. To quantify cells grown on seeded scaffolds, optical density values measured for each sample were plotted on the standard curve, gaining the corresponding cell number.

For SEM analysis, the samples were processed as described in Section 4.2.3.

### 4.4. In Vivo Biocompatibility Study

#### 4.4.1. Subcutaneous Implant

Hybrid OxPVA scaffold biocompatibility was verified through a heterotopic implant in mice [58]. Preliminarily, OxPVA/ChS discoidal samples (8 mm diameter; 2.5 mm in thickness) were prepared using a biopsy punch and disinfected by soaking in a 2% penicillin/streptomycin solution in PBS under UV light (15 min/side). Hence, twelve female Balb/c mice (12 weeks old; mean weight 19.8 ± 1.3 g) were anesthetized by gaseous anaesthesia (isoflurane/oxygen, in mixture) and, after shaving and disinfecting the dorsal cutis, a median dorsal pouch (10 mm in length) was created by a No. 10 surgical blade (Becton-Dickinson, Franklin Lakes, NJ, USA). The scaffolds were carefully placed and fixed to the latissimus dorsi muscle using Tycron 4/0 sutures to avoid eventual dislocation/migration, while the skin was sutured by absorbable Novosyn 4/0 stitches. The animals were administered antibiotic (Bytril, 5 mg/kg) and anti-inflammatory (Rimadil, 5 mg/kg) therapy for 5 days after surgery. In the following period, they were housed in a temperature-controlled facility and were given laboratory rodent diet and water *ad libitum*.

Euthanasia occurred after 14 days from by carbon dioxide asphyxiation; tissues were dissected and the implant-site was preliminarily observed for possible evidence of inflammation-related signs. Thereafter, the retrieved scaffolds, together with the surrounding tissues. were adequately fixed and investigated by histology and immunohistochemistry to unravel the biological response triggered in vivo. The ultrastructural evaluation of the scaffolds surface was also performed by SEM.

#### 4.4.2. Histological Analyses

Graft integration and the inflammatory infiltrate at the graft–host interface was assessed by Hematoxylin and Eosin staining, whereas scaffold encapsulation by fibroconnective tissue was evaluated using Masson’s trichrome staining.

Samples were first fixed in 10% formalin for one week, paraffin-embedded, cut into 5 μm-thick sections, dewaxed, and rehydrated with a series of ethanol (Vetrotecnica) solutions (99%, 95%, 70%) before being stained. Regarding Haematoxylin and Eosin staining, the dewaxed and rehydrated sections were immersed in haematoxylin for 8 min, tap water for 5 min, and eosin for 1 min. The sections were then dehydrated in a series of ethanol (95% for 45 s and 100% for 3 min (×2)), and finally immersed in xylene. Each section was then mounted with Eukitt (Merck LifeScience, Darmstadt, Germany). Masson’s trichrome staining occurred using the Masson trichrome staining kit (Bio-Optica, Milano, Italy), following the manufacturer’s instructions. Briefly, the rehydrated sections were exposed to Weigert’s iron haematoxylin (A solution) + Weigert’s iron haematoxylin (B solution) (for 10 min); without washing, the slides were drained and picric acid alcoholic stable solution (C solution) was dripped on the sections and allowed to act (for 4 min). Thus, after a quick wash in distilled water (3–4 s), Ponceau acid fuchsin according to Masson (D solution) was added to the section (for 4 min). Following a wash in distilled water, phosphomolybdic acid solution was added to the section (E solution) (for 10 min). Without washing, the slides were drained and a light green solution according to Goldner (reagent F) was left to act (for 5 min). Finally, the sections were washed in distilled water, dehydrated, and cleared in xylene as previously described, and mounted with Eukitt (Merck LifeScience).

#### 4.4.3. Immunohistochemical Investigation

The host immune response towards OxPVA/Chs scaffolds implantation was characterized by the immunolocalization of lymphocyte T (CD3+) and monocyte/macrophage (F4/80+) infiltrates at the graft boundaries. Formalin-fixed and paraffin-embedded sections (3 µm thickness) were dewaxed and rehydrated as described in Section 4.4.2; hence, immunohistochemical reactions were performed by Dako Autostainer/Autostainer Plus (Dako, Milan, Italy) with the following primary antibodies diluted in PBS: anti-CD3 (polyclonal rabbit anti-CD3, A0452, Dako) (1:500) and anti-F4/80 (polyclonal rabbit anti-F4/80 (M-17)-R, sc-26643-R, Santa Cruz Biotechnology) (1:1000) to label lymphocytes and monocytes/macrophages, respectively. The binding between the primary antibody and specific antigens was then revealed using a labelled polymer (EnVision™ FLEX-HRP; Dako) and 3,3′-diaminobenzidine (EnVision™ FLEX Substrate buffer + DAB + Chromogen; Dako). Meanwhile, negative controls were developed without incubation with primary antibodies.

#### 4.4.4. Ultrastructural Evaluation by SEM

After sample retrieval, the analysis of scaffold ultrastructure was performed by processing specimens as described in Section 4.2.3 for SEM investigation.

### 4.5. Statistical Analysis

Data were expressed as the mean ± standard deviation (SD) of six different replicates (five images for porosity analysis based on SEM photomicrographs). Statistical analysis was performed by the one-way analysis of variance (ANOVA) and Tukey’s post hoc test for multiple comparisons. Differences among experimental groups were considered statistically significant at *p* < 0.05. Statistical analyses were performed by Prism 8.1.0 (GraphPad Software).

## 5. Conclusions

According to in vitro/in vivo experimental evidence, the topographic modulation of OxPVA scaffolds surfaces by coupling with a chitosan bioactive layer proved safe and effective; specifically, different chitosan sponges were here compared, including ChS as such, Ch+EAK, Ch+EAK-IKVAV, and Ch+EAK-YIGSR.

ChS, even without the presence of SAPs, showed a certain ability in supporting cell adhesion and proliferation; this attitude is mainly mediated by sponge ultrastructure, depending on the fabrication method. The presence of bioactive SAPs did not interfere with the cell colonization of chitosan sponges; moreover, it was demonstrated that SAPs chain lengths can modulate chitosan’s fine organization: longer chains (+EAK-IKVAV, +EAK-YIGSR) lead to larger interconnected pores than shorter sequences (+EAK). Interestingly, modulating the length of bioactive peptides can tune the mechanical properties of chitosan. It will be enlightening for future pre-clinical studies to assess the bioactive role of the peptides regarding both the impact of different NC lumen ultrastructures and the specific bioactive role of the sequences.

OxPVA/ChS-based scaffolds may have great potential for biomedical applications; however, further research is required to identify the ideal equilibrium between stimuli (3D organization, bioactive sequences length/type). This study lays the basis for future vanguard hybrid scaffolds supporting structural and functional nerve recovery, including nerve conduits for long-gap reconjunction. Additionally, other possible end-use destinations cannot be excluded through both varying and combining different SAPs.

## Figures and Tables

**Table 1:** Mechanical properties of ChS, ChS+EAK, ChS+EAK-IKVAV, and ChS+EAK-YIGSR scaffolds: compressive modulus € and maximum stress (σ_max_) reported as mean value ± standard deviation.

Scaffold	E (kPa)	σ_max_ (kPa)
ChS	40.4 ± 3.1	16.8 ± 1.0
ChS+EAK	31.1 ± 2.1	11.9 ± 0.6
ChS+EAK-IKVAV	30.9 ± 1.9	7.3 ± 0.4
ChS+EAK-YIGSR	30.6 ± 2.0	7.2 ± 0.4

**Table 2:** Statistical analysis on mechanical properties of ChS, ChS+EAK, ChS+EAK-IKVAV, and ChS+EAK-YIGSR scaffolds: Tukey’s HSD results.

Pairwise Comparisons	E—Remarks	σ_max_—Remarks
ChS vs. ChS+EAK	** *p* < 0.01	** *p* < 0.01
ChS vs. ChS+EAK-IKVAV	** *p* < 0.01	** *p* < 0.01
ChS vs. ChS+EAK-YIGSR	** *p* < 0.01	** *p* < 0.01
ChS+EAK vs. ChS+EAK-IKVAV	Not Significant	** *p* < 0.01
ChS+EAK vs. ChS+EAK-YIGR	Not Significant	** *p* < 0.01
ChS+EAK-IKVAV vs. ChS+EAK-YIGSR	Not Significant	Not Significant

** *p* < 0.01

## Data Availability

Not applicable.

## Supplementary Materials

The following supporting information can be downloaded at: https://www.mdpi.com/article/10.3390/ijms232012059/s1.
