# Supplementary material for: Development of Two-Layer Hybrid Scaffolds Based on Oxidized Polyvinyl Alcohol and Bioactivated Chitosan Sponges for Tissue Engineering Purposes
Source: Int J Mol Sci. 2022 Oct 11;23(20):12059. doi: 10.3390/ijms232012059 (PMC9603651; doi:10.3390/ijms232012059)
Supplement: Supplementary file 1 [file ijms-23-12059-s001.zip › ijms-1910811-supplementary.pdf]

Supplementary Material

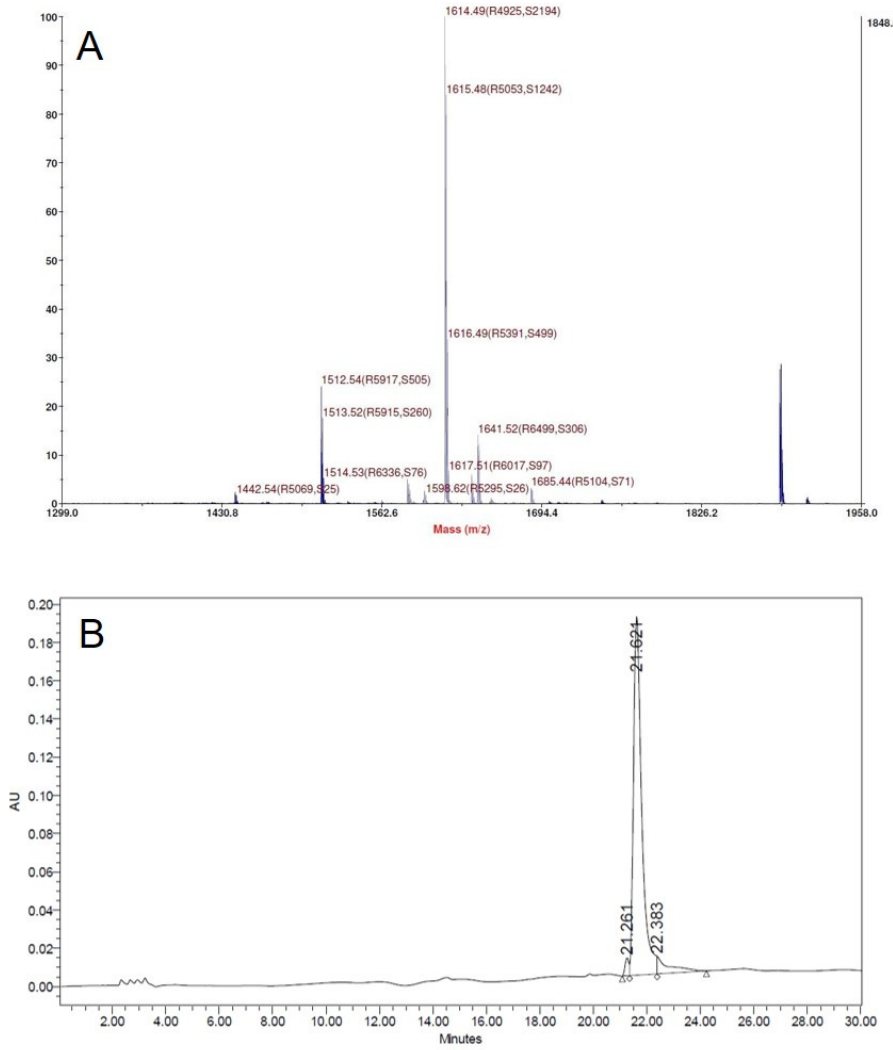

Figure S1. (A) MALDI-TOF spectrum of crude peptide EAK: experimental mass = 1614.520 Da; theoretical mass = 1614.79 Da. (B) Analytical chromatogram of purified EAK (conditions: Vydac C_{18} column (5 μm, 300 Å, 4.6 × 250 mm, Grace), eluent A: 0.05% TFA in H_2O; eluent B: 0.05% TFA in CH_3CN; gradient: from 5 to 20 % di B in 30 min, flow rate: 1 mL/min; detector: 214 nm).

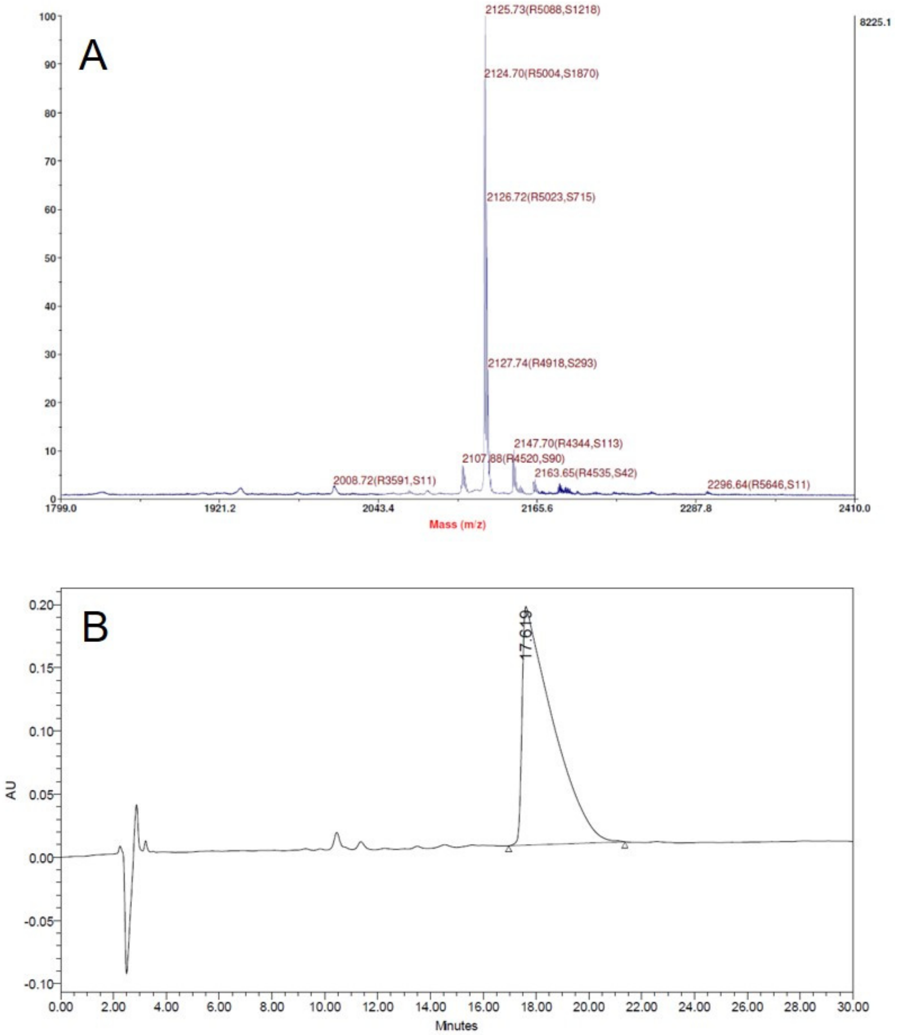

Figure S2. (A) MALDI-TOF spectrum of crude peptide EAK-IKVAV: experimental mass = 2125.73 Da; theoretical mass = 2125.48 Da. (B) Analytical chromatogram of purified EAK-IKVAV (conditions: Nova-Pak HR C_{18} column (4 µm, 60 Å, 3.9 × 300 mm, Waters), eluent A: 0.05% TFA in H_2O; eluent B: 0.05% TFA in CH_3CN; gradient: from 15 to 30% di B in 30 min, flow rate: 1 mL/min; detector: 214 nm).

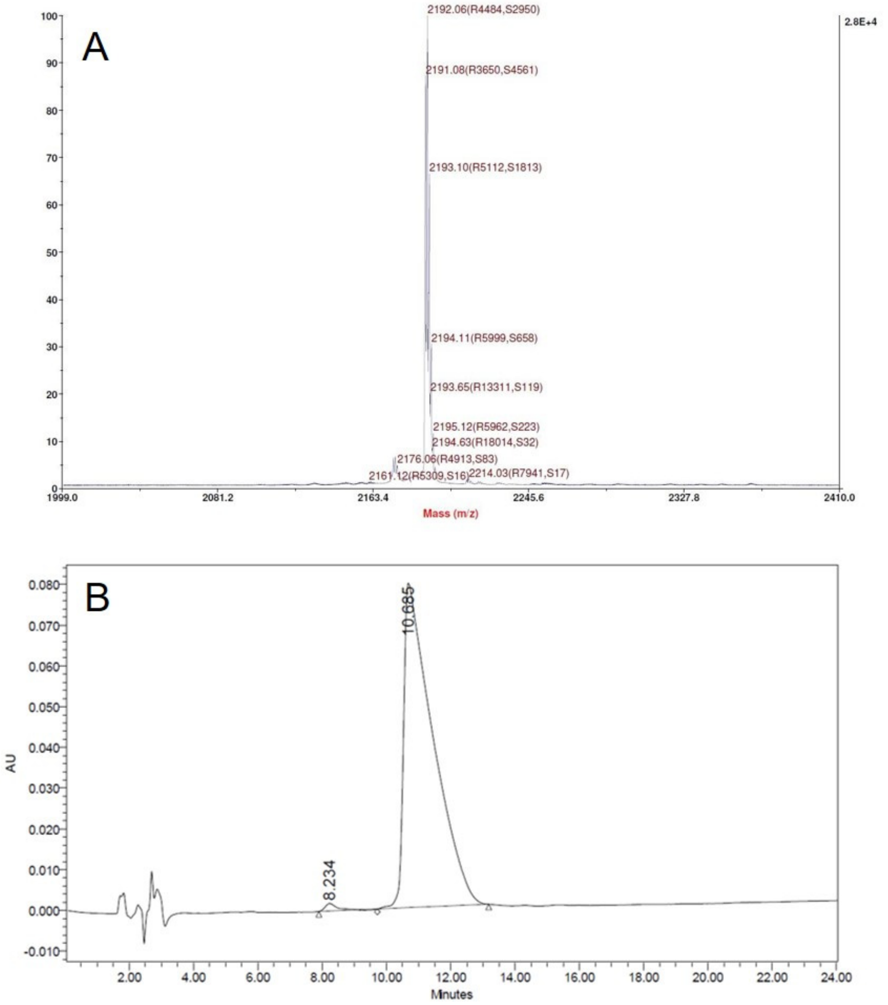

Figure S3. (A) MALDI-TOF spectrum of crude peptide EAK-YIGSR: experimental mass = 2192.06 Da; theoretical mass = 2191.45 Da. (B) Analytical chromatogram of purified EAK-YIGSR (conditions: Nova-Pak HR C_{18} column (4 µm, 60 Å, 3.9 × 300 mm, Waters), eluent A: 0.05% TFA in H_2O; eluent B: 0.05% TFA in CH_3CN; gradient: from 18 to 26 % di B in 24 min, flow rate: 1 mL/min; detector: 214 nm).
